# Housekeeping genes tend to show reduced upstream sequence conservation

**DOI:** 10.1186/gb-2007-8-7-r140

**Published:** 2007-07-13

**Authors:** Domènec Farré, Nicolás Bellora, Loris Mularoni, Xavier Messeguer, M Mar Albà

**Affiliations:** 1 Centre for Genomic Regulation, Dr Aiguader 88, Barcelona 08003, Spain; 2 Universitat Pompeu Fabra, Dr Aiguader 88, Barcelona 08003, Spain; 3 Fundació Institut Municipal d'Investigació Mèdica, Dr Aiguader 88, Barcelona 08003, Spain; 4 Universitat Politècnica de Catalunya, Jordi Girona 1-3, Barcelona 08034, Spain; 5 Catalan Institution for Research and Advanced Studies, Pg Lluis Companys 23, Barcelona 08010, Spain

## Abstract

**Background:**

Understanding the constraints that operate in mammalian gene promoter sequences is of key importance to understand the evolution of gene regulatory networks. The level of promoter conservation varies greatly across orthologous genes, denoting differences in the strength of the evolutionary constraints. Here we test the hypothesis that the number of tissues in which a gene is expressed is related in a significant manner to the extent of promoter sequence conservation.

**Results:**

We show that mammalian housekeeping genes, expressed in all or nearly all tissues, show significantly lower promoter sequence conservation, especially upstream of position -500 with respect to the transcription start site, than genes expressed in a subset of tissues. In addition, we evaluate the effect of gene function, CpG island content and protein evolutionary rate on promoter sequence conservation. Finally, we identify a subset of transcription factors that bind to motifs that are specifically over-represented in housekeeping gene promoters.

**Conclusion:**

This is the first report that shows that the promoters of housekeeping genes show reduced sequence conservation with respect to genes expressed in a more tissue-restricted manner. This is likely to be related to simpler gene expression, requiring a smaller number of functional *cis*-regulatory motifs.

## Abstract 2

Mammalian housekeeping genes show significantly lower promoter sequence conservation, especially upstream of position -500 with respect to the transcription start site, than genes expressed in a subset of tissues.

## Background

The correct functioning of multicellular organisms depends on a complex orchestration of gene regulatory events, which ensure that genes are expressed at the right time, place and level. Much of this regulation occurs at the level of gene transcription, and is mediated by specific interactions between transcription factors and *cis*-regulatory DNA motifs. Regulatory motifs concentrate in sequences upstream of the transcription start site (TSS), the region known as the gene promoter (for a recent review, see [1]).

Changes in gene expression patterns can cause important phenotypic modifications. Mutations in *cis*-regulatory motifs can alter the binding affinity of transcription factors and affect the expression of a gene. However, the evolutionary dynamics of promoter sequences are still poorly understood. A commonly used approach to assess the existence of evolutionary constraints and identify regulatory motifs is the identification of conserved non-coding sequences across orthologues. This rationale is behind several described 'phylogenetic footprinting' methods to discover functional regulatory sequences [2-4].

Contrary to coding sequences, gene expression regulatory sequences do not have very well defined boundaries. A region spanning approximately 100 base-pairs (bp) upstream of the TSS, known as the basal promoter, plays a fundamental part in the assembly of the transcription initiation complex. Further upstream regulatory sequences are of variable length depending on the particular gene [1]. Nevertheless, a recent study has shown that, at distances longer than 2 Kb from the TSS, the similarity between orthologous promoters drastically drops, indicating that most of the functional elements concentrate in the 2 Kb promoter region [5]. In accordance, about 85% of the known mouse transcription regulatory motifs are located within 2 Kb of the gene promoter region [6] and functional assays have shown that a region spanning -500 to +50 relative to the TSS region is sufficient to drive transcription in cultured cells for most human genes [7].

Promoter sequence comparisons across different species have shed light on the different constraints exhibited by promoters of different types of genes. In particular, it has been observed that the promoters of genes encoding regulatory proteins, such as transcription factors and/or developmental proteins, tend to show remarkably strong sequence conservation [8,9], suggesting that the expression of this class of genes requires a relatively large amount of *cis*-regulatory motifs.

Another important factor that may be related to promoter sequence conservation is the number of tissues in which a gene is expressed. In the adult organism, some genes show high tissue-specificity while others show little or no tissue expression restrictions (ubiquitous expression). The effect of expression breadth on promoter conservation has not been addressed previously. Here we provide evidence that, in mammals, the simple expression patterns exhibited by housekeeping genes - expressed in all or nearly all tissues - are often associated with limited promoter sequence conservation, while tissue expression restrictions are associated with increasingly high promoter conservation. This defines a new important property of mammalian gene promoters.

## Results

### Divergence of orthologous human and mouse promoter sequences

The promoters of different genes exhibit varying degrees of sequence divergence [8-10]. In genes from nematodes [11] and yeast [12], the level of promoter sequence divergence is positively correlated with the evolutionary rate of the encoded protein. An interesting question is whether such a correspondence also exists in mammals. We collected human and mouse orthologous promoters (6,698 pairs, 2 Kb from the transcription start site) and applied different measures of sequence divergence. We aimed at quantifying promoter sequence divergence, evaluating the strength of selection and identifying any significant relationship between the divergence of promoter and coding sequences.

First, we calculated the fraction of the promoter sequence that failed to align between human and mouse orthologues. We used the local pairwise sequence alignment program described in Castillo-Davis *et al*. [11], which provides a score, d_SM _(shared motif divergence), that corresponds to the fraction of non-aligned sequence. The average value was 0.701, which means that, on average, 29.9% of the 2 Kb promoter sequence was successfully aligned. On the promoter alignments we estimated the number of nucleotide substitutions per site using PAML [13]. This promoter substitution rate, which we term Kp, was, on average, 0.334 substitutions per site.

Next we estimated the synonymous (Ks) and non-synonymous (Ka) substitution rates of the corresponding gene coding sequences using PAML. In mammals, Ks can be used to account for the background mutation level. Ka, on the contrary, corresponds to changes at the amino acid level and reflects the strength of selection on the protein. In the orthologous dataset, the average Ks was 0.709 and the average Ka 0.084. The approximately two-fold difference between Kp and Ks (0.334 and 0.709, respectively) indicates stronger negative or purifying selection in the evolution of promoter sequences with respect to synonymous sites in coding regions.

We subsequently addressed the question of whether the level of promoter sequence divergence is related to the evolutionary rate in the corresponding coding sequence in mammals. Interestingly, we found a modest although significant positive correlation between the promoter divergence (d_SM_) and the coding sequence substitution rate (d_SM _and Ka, r = 0.20, *p *< 10^-58^; d_SM _and Ka/Ks, r = 0.14, *p *< 10^-29^; d_SM _and Ks, r = 0.18, *p *< 10^-48^). That is, in general, proteins that showed high divergence between human and mouse (high Ka or Ka/Ks) showed a tendency to be encoded by genes with reduced promoter sequence conservation.

### Gene expression breadth

**Figure 1:**
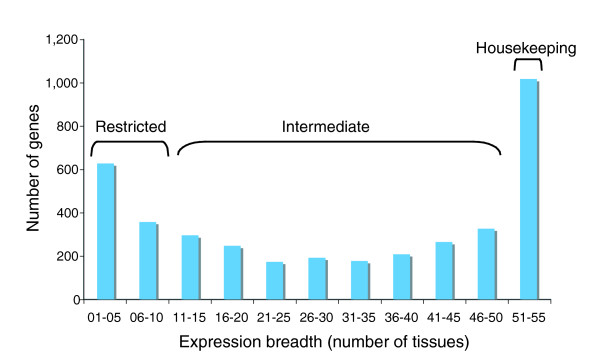
Mouse tissue expression distribution. We define three groups: low expression breadth (Restricted; 1-10 tissues), intermediate expression breadth (Intermediate; 11-50 tissues), high expression breadth (Housekeeping; 51-55 tissues).

We used mouse transcriptome microarray data from Zhang *et al*. [14] to classify the previously defined genes into different groups according to their expression in 55 mouse organs and tissues (see Supplementary table S5 in Additional data file 1). The orthologous dataset with expression data contained 3,893 genes. The tissue distribution profile in five-tissue bins (Figure 1) showed a bimodal shape with a moderate excess of genes expressed in a few tissues and a more acute excess of genes expressed in a very large number of tissues. Genes with expression restricted to 1-10 tissues were classified as 'restricted' (986 genes), those with ubiquitous or nearly ubiquitous expression (51-55 tissues) as 'housekeeping' (HK; 1,018 genes), and the rest, expressed in 11-50 tissues, as 'intermediate' (1,889 genes).

We compared d_SM_, Kp, Ka and Ks values for genes classified in the three different expression groups (Table 1). We observed that the average d_SM _score, which corresponds to the fraction of the 2 Kb promoter that cannot be aligned, consistently increased with the expression breadth. The average d_SM _in HK genes was 0.732 (26.8% promoter conservation), whereas in genes with 'restricted' expression it was 0.688 (31.2% promoter conservation). The d_SM _values were significantly different between HK genes and the other non-HK groups (Wilcoxon-Mann-Whitney and Kruskal-Wallis tests, *p *< 10^-5^). The nucleotide substitution rate within aligned regions, Kp, was, instead, not significantly different across the different datasets. Kp also showed decreased variability with respect to Ks, with about three times lower standard deviation values (Table 1). In contrast to promoter divergence, both Ka and Ka/Ks in coding sequences were significantly lower in HK genes than in the other groups (Table 1). In fact, we observed a negative correlation between expression breadth and Ka (r = -0.31, *p *< 10^-87^), in accordance with previous results [15,16]. Therefore, while at the promoter level the constraints appeared to be weaker in HK genes than in the rest of the genes, at the level of the protein sequence the situation was reversed.

**Table 1:** Sequence divergence versus tissue expression breadth

No. of tissues	N (total = 3,893)	d_SM_	Kp	Ka	Ks	Ka/Ks
01-10	986	0.688	0.337	**0.107**	**0.733**	**0.150**
		0.735	0.328	0.084	0.673	0.119
		0.221	0.110	0.093	0.299	0.122
11-50	1,889	0.701	0.333	**0.079**	0.708	0.116
		0.752	0.328	0.058	0.633	0.089
		0.216	0.093	0.073	0.307	0.103
51-55	1,018	**0.732**	0.328	**0.050**	**0.639**	**0.079**
		0.791	0.323	0.031	0.572	0.054
		0.208	0.079	0.057	0.305	0.085
	*p *value (K-W test)	<10^-5^	0.226	<10^-75^	<10^-18^	<10^-62^

N, number of genes; d_SM_, promoter divergence (see text); Kp, promoter substitution rate; Ka, non-synonymous substitution rate; Ks, synonymous substitution rate. Mean (top), median (middle), and standard deviation (bottom) are indicated for each variable. Numbers in bold indicate significant differences at *p *< 0.001 in each expression group with respect to the rest (two-sample Wilcoxon-Mann-Whitney test). The last row shows the *p *value of Kruskal-Wallis (K-W) test that evaluates differences between the three tissue expression breadth groups.

Additional support for the results was obtained using human gene expression data. We mapped the orthologous genes to the eVOC database (anatomical system and cell type) [17], based on expressed sequence tag data, and to Gene Atlas [18]. The results obtained using these datasets were in strong agreement with the results presented in Table 1 (see Supplementary tables S1, S2 and S3, respectively, in Additional data file 1). That is, the fraction of human genes with the broadest tissue expression (HK genes) always showed significantly higher promoter divergence values.

**Figure 2:**
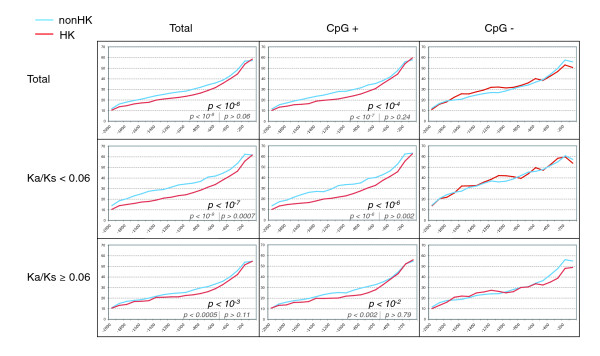
Promoter sequence conservation in HK and non-HK genes. The x-axis shows 100 nucleotide bins along 2 Kb upstream of the TSS. The y-axis shows percent conservation ((1 - d_SM_) × 100). Genes were grouped according to the presence or absence of a CpG island and Ka/Ks values. Significant *p *values for 2 Kb promoter sequence divergence comparisons are indicated below the curves. Beneath these, the *p *values obtained for regions -2,000 to -500 (left), and -500 to the TSS (right), are given in smaller font size.

The next question we addressed was whether the reduced sequence conservation observed in HK genes was uniformly distributed along the 2 Kb upstream sequence or, alternatively, it could be mapped to a particular region of the promoter. Considering the complete 2 Kb sequences, d_SM _differences between HK and non-HK datasets were significant at *p *< 10^-6 ^(Wilcoxon-Mann-Whitney test). Then, we calculated the average sequence conservation (1 - d_SM_) in 100 nucleotide overlapping sequence windows (bins) along the 2 Kb promoter sequence in HK and non-HK genes (Figure 2, top row, left). We found that the region spanning from the TSS to position -100 showed the highest level of sequence conservation (average 1 - d_SM _0.576, or 57.6% promoter conservation). Further upstream, the sequence conservation gradually dropped, with a stronger decay in HK than in non-HK genes (Figure 2, top row, left). If we considered only the proximal promoter region, from the TSS to position -500, we did not detect statistically significant differences (*p *= 0.0633). However, using the region from the TSS to -600, differences became significant at *p *< 0.05 (*p *= 0.0195). On the other hand, when we considered the distal promoter region only, from -500 to -2,000, the gap between the two types of sequences regarding promoter divergence increased (*p *< 10^-8^). Therefore, we concluded that the observed lower promoter sequence conservation of HK genes concentrated in regions upstream from position -500.

### Functions of encoded gene products

Our data show that HK genes contained poorly conserved promoters, particularly in the promoter distal part (upstream from -500). Other studies reported differences in the conservation of promoter sequences in relation to the function of the protein [8,9]. As HK genes encode proteins with biased function composition [19,20], we measured the over- and under-representation of different Gene Ontology (GO) terms [21] in the group of HK genes. We also assessed whether the functional biases in HK genes could alone explain the differences observed in promoter sequence conservation.

We determined which GO classes were over- or under-represented among HK genes (*p *< 0.01, χ^2 ^test), using the 'molecular function', 'biological process', and 'cellular component' classification systems (Supplementary table S4 in Additional data file 1). As expected, an important fraction of the classes statistically over-represented among HK genes showed significantly high promoter sequence divergence. For example, in genes classified as 'structural constituent of ribosome', and 'mitochondrion' the average promoter sequence conservation was only 23% (d_SM _= 0.77). On the other hand, many classes under-represented among HK genes showed significantly high promoter sequence conservation (low d_SM_). For example, genes annotated as 'transcription factor activity' or 'nervous system development' showed an average promoter conservation of 42% (d_SM _= 0.58), and genes annotated as 'cell differentiation' showed an average promoter conservation of 43% (d_SM _= 0.57).

Given the promoter sequence divergence differences among gene functional classes, one possibility was that the functional class bias in HK genes could fully explain the differences found between HK and non-HK genes. For this reason we tested whether there were any d_SM _differences between HK and non-HK genes within the same GO class. For statistical robustness we considered only GO classes with a minimum of 150 genes (22 classes; Table 2). In 19 of these classes, the average d_SM _of HK genes was higher than that of non-HK genes. For example, transcription factors with HK expression had an average d_SM _of 0.673 (32.7% promoter conservation), while those with no HK expression had an average d_SM _of 0.602 (39.8% promoter conservation). Of the 19 classes, 9 showed significant d_SM _differences between HK and non-HK genes (*p *< 0.05). On the other hand, in the three classes with higher average d_SM _scores in non-HK than in HK genes the differences were not significant (*p *> 0.64). Therefore, we concluded that the promoter sequence divergence differences between HK and non-HK genes were essentially maintained within the different GO classes.

**Table 2:** Average promoter divergence values (d_SM_) for HK and non-HK genes classified in different GO classes

		All			CpG+			CpG-		
				
GO term	Description	N	d_SM _(HK)	d_SM _(nonHK)	N	d_SM _(HK)	d_SM _(nonHK)	N	d_SM _(HK)	d_SM _(nonHK)
Molecular function										
GO:0000166	Nucleotide binding	464	0.727	0.699	**363**	**0.732**	**0.698**	101	0.684	0.700
GO:0004872	Receptor activity	259	0.734	0.675	131	0.747	0.656	128	0.655	0.692
GO:0004871	Signal transducer activity	440	0.689	0.658	246	0.692	0.656	194	0.663	0.661
GO:0003700	Transcription factor activity	183	0.673	0.602	113	0.657	0.600	70	0.766	0.605
GO:0043169	Cation binding	485	0.711	0.671	**308**	**0.732**	**0.670**	177	0.582	0.671
										
Biological process										
GO:0044249	Cellular biosynthesis	**256**	**0.765**	**0.735**	**183**	**0.781**	**0.729**	73	0.629	0.741
GO:0045184	Establishment of protein transport	162	0.720	0.737	138	0.723	0.731	24	0.677	0.760
GO:0007049	Cell cycle	188	0.697	0.706	152	0.703	0.724	36	0.656	0.646
GO:0019538	Protein metabolism	**700**	**0.748**	**0.703**	**523**	**0.755**	**0.698**	177	0.682	0.713
GO:0044260	Cellular macromolecule metabolism	**761**	**0.748**	**0.705**	**560**	**0.755**	**0.700**	201	0.686	0.713
GO:0050874	Organismal physiological process	**292**	**0.795**	**0.681**	**109**	**0.813**	**0.675**	183	0.756	0.685
GO:0009605	Response to external stimulus	209	0.676	0.711	85	0.758	0.699	**124**	**0.538**	**0.718**
GO:0007166	Cell surface receptor linker signal transduction	221	0.683	0.626	113	0.659	0.645	108	0.762	0.609
GO:0048513	Organ development	**214**	**0.677**	**0.566**	**103**	**0.699**	**0.528**	111	0.633	0.598
GO:0009653	Morphogenesis	**262**	**0.679**	**0.584**	**132**	**0.685**	**0.549**	130	0.664	0.615
GO:0009607	Response to biotic stimulus	166	0.761	0.723	**74**	**0.783**	**0.686**	92	0.680	0.745
GO:0007165	Signal transduction	563	0.684	0.656	342	0.687	0.668	221	0.666	0.643
										
Cellular component										
GO:0005739	Mitochondrion	171	0.785	0.756	148	0.780	0.770	23	0.869	0.707
GO:0005737	Cytoplasm	**773**	**0.756**	**0.719**	**579**	**0.759**	**0.727**	194	0.728	0.707
GO:0005783	Endoplasmic reticulum	**153**	**0.791**	**0.713**	**112**	**0.776**	**0.712**	**41**	**0.881**	**0.713**
GO:0005576	Extracellular region	219	0.653	0.621	77	0.718	0.591	142	0.523	0.635
GO:0005886	Plasma membrane	**373**	**0.720**	**0.661**	**189**	**0.735**	**0.656**	184	0.663	0.666

Entries in bold are those that have a significantly different d_SM _distribution (*p *< 0.05). The number of genes (N) is indicated for each GO class. Results for CpG+ and CpG- genes are shown.

### CpG island content and coding sequence evolutionary rate

The promoters of HK genes are rich in CpG islands [22-25]. This could potentially influence the level of conservation of promoter sequences. Therefore, we divided the gene dataset into genes containing CpG islands (CpG+) and genes not containing CpG islands (CpG-), according to the presence or absence of a CpG island in the region -100 to +100 (see Materials and methods), and analyzed the two groups separately. Of the mouse genes, 65% were classified as CpG+ (91% of the human orthologs of these were also CpG+). Among the genes classified as HK, this number went up to 88%. The length of CpG islands was not significantly different in HK and non-HK genes.

Within CpG+ genes, we observed the previously described positive relationship between promoter sequence divergence (d_SM_) and expression breadth. HK genes (expressed in 51-55 tissues) had an average d_SM _of 0.739, whereas genes expressed in an intermediate number of tissues (11-50) and those with restricted expression (1-10 tissues) had average d_SM _scores of 0.708 and 0.679, respectively. These scores are comparable to those obtained previously (Table 1) and the differences between HK and non-HK genes were highly significant (*p *< 10^-4^; Figure 2, top row, middle). Similar results were obtained with other gene expression datasets (Figures S1, S2 and S3 in Additional data file 2).

In contrast, in CpG- genes the differences between HK and non-HK genes were smaller, and did not reach statistical significance in the mouse gene dataset (Figure 2, top row, right). Indeed, HK genes that did not contain CpG islands (12% of the HK genes) showed average promoter sequence divergence similar to that of non-HK genes (around 0.69). Thus, this minority of HK genes with no CpG islands appeared to have increased sequence evolutionary constraints in relation to the rest of the HK genes.

We also assessed if the presence or absence of CpG islands influenced d_SM _differences between HK and non-HK genes within the same GO class. In CpG+ genes the differences between HK and non-HK genes were even more marked than in the complete dataset, and three additional GO functions showed statistical differences (Table 2). In CpG- genes, instead, the differences between HK and non-HK genes per GO class were, in almost all cases, not significant.

We had previously described a positive correlation between the non-synonymous substitution rate, Ka (or Ka/Ks), and promoter sequence divergence (d_SM_). That is, many rapidly evolving coding sequences were associated with poorly conserved promoters. This seemed at first to contradict the finding that HK genes, with typically low Ka values, tended to have highly divergent promoters. To unravel the effect of coding sequence evolutionary rate and expression breadth in promoter sequence evolution, we divided the gene dataset into two groups, genes with Ka/Ks < 0.06, a fraction representing about one-third of the genes and highly enriched in HK genes, and the rest of the genes, with Ka/Ks ≥ 0.06.

The first observation was that, according to the general correlation, genes with more slowly evolving coding sequences (Ka/Ks < 0.06) showed higher promoter conservation than those with Ka/Ks ≥ 0.06 (average d_SM _of 0.663 and 0.722, respectively). However, this was mostly due to genes that were not HK genes (Figure 2, middle row, left), which explained the apparent contradiction mentioned before. Among genes with Ka/Ks < 0.06, the average d_SM _was 0.72 for HK genes, but 0.65 for non-HK genes. Not surprisingly, we found that the previously observed correlation between d_SM _and Ka/Ks was more relevant in non-HK genes (r = 0.17, *p *< 10^-19^) than in HK genes (r = 0.10, *p *< 0.002).

### *Cis*-regulatory motif content in housekeeping gene promoters

The differences in promoter sequence divergence associated with expression tissue distribution are likely to reflect the presence of different functional regulatory motifs in genes with diverse expression patterns. Among the expression groups previously defined (restricted, intermediate and HK) only the HK gene group probably represents a rather homogeneous class from a gene expression regulatory perspective. Other groups include genes that are active in diverse tissues and that are likely to be regulated by very different factors. We thus investigated whether the promoters of HK genes were enriched in specific transcription factor binding motifs.

In the first place, we mapped all experimentally verified transcription factor binding sites (TFBSs) from TRANSFAC [26] in the human and mouse promoter sequences. We observed that approximately 75% of mapped TFBSs fell into conserved regions, which only occupy approximately 30% of the sequence analyzed. However, as only less than 2% of the genes in the dataset contained known TFBSs, we could not infer any statistically significant biases from these data. For this reason, we decided to use motifs predicted by weight matrices representing known TFBSs. We performed separate analysis with the vertebrate TFBS weight matrix collections available from TRANSFAC and PROMO [27]. We identified nine motifs that were consistently over-represented in the aligned parts of HK gene promoters using the two weight matrix datasets (χ^2 ^test, *p *< 10^-5^; Table 3). The motifs were recognized by particular transcription factors or families of transcription factors, according to data in TRANSFAC and PROMO. Among them were commonly found regulators such as Sp1, or members of the ATF (activating transcription factor) family. We also analyzed HK motif over-representation separately in aligned regions located either downstream or upstream of position -500. Whereas in the region from the TSS to -500 the nine distinct motifs became even more strongly over-represented than in the 2 Kb promoter, in the more distal promoter region, upstream of -500, four of the motifs - ATF, CREB, NRF1/2 and USF - were no longer significant. We next determined the expression class of the transcription factors that could bind to the nine motif types, using the previously defined three expression groups. Importantly, all transcription factors showed HK or intermediate expression patterns (Table 3), and none showed tissue-restricted expression, which is consistent with a putative role in the regulation of HK genes. Therefore, we could define a group of factors that, mainly through interactions with HK proximal promoter regions, are likely to play important roles in the maintenance of adequate levels of expression of this type of genes.

**Table 3:** Transcription factors with predicted binding motifs over-represented in HK gene promoters

Transcription factor	Description	Expression breadth
AHR and ARNT	Aryl hydrocarbon receptor; it can interact with ARNT (AHR:ARNT heterodimer)	INT
ATF family	Activating transcription factor	HK
CREB family	cAMP responsive element binding protein	INT
E2F family	E2F transcription factor	INT and HK
HIF1A	Hypoxia inducible factor 1, alpha subunit; as AHR, it can interact with ARNT	HK
MYC and MAX	Proto-oncogene protein c-myc and MYC associated factor X; they can form MYC:MAX heterodimers	INT and HK
NRF1 and NRF2	Nuclear respiratory factor 1 and 2	INT and HK
SP1	SP1 transcription factor	HK
USF	Upstream transcription factor (USF1 and USF2)	INT

HK, housekeeping; INT, intermediate.

## Discussion

In this work we present the first evidence, at least to our knowledge, of a relationship between promoter sequence divergence and gene expression breadth. We have observed that the promoters of HK genes tend to be less conserved than those of non-HK genes, especially in the distal promoter region, upstream of position -500. Given the strong conservation of HK gene expression patterns across organisms [28], high promoter sequence divergence is likely to reflect weak functional constraints rather than sequence diversification driven by the acquisition of new functionalities. These observations raise the interesting possibility that HK genes have shorter functional promoters. Interestingly, other features of HK genes tend to shortness; in particular, they have been described to have shorter coding, intronic, and intergenic sequences [29-31]. As a consequence, and with the exception of plants [32], transcripts of HK genes tend to be short. One hypothesis put forward to explain this observation is selection for economy in transcription and translation [30,31]. An alternative hypothesis, called 'genome design', is that tissue-specific genes require a greater amount of non-coding DNA due to their more complex regulation [29]. Our results show that HK genes contain more divergent distal promoter sequences than non-HK genes. In line with the 'genome design' hypothesis, this may be due to their relatively simple expression patterns, requiring less regulatory sequences.

In mammals, conservation of a gene's upstream sequence is related to the function of the encoded protein [8,9]. Iwama and Gojobori [9] found that genes encoding transcription factors and developmental proteins showed high gene upstream sequence conservation. Similarly, Lee *et al*. [8] showed that genes involved in complex and adaptative processes, such as development, cell communication, neural function, and signaling, were associated with higher promoter sequence conservation despite their relative recent emergence during evolution. On the contrary, genes involved in basic processes, such as metabolism and ribosomal function, contained poorly conserved promoters. Our study is consistent with these findings, as the former genes are under-represented in HK genes, while the later are over-represented. However, by directly relating promoter conservation to mode of expression, we are able to propose a more direct explanation for the differences in promoter sequence conservation between genes that perform basic housekeeping functions, and which are simply regulated, and genes that are important for tissue- or organ-specific processes, which may require a more complex regulation. In addition, function alone cannot explain the differences across genes, as the reduced promoter sequence conservation in HK genes with respect to non-HK genes is essentially maintained within different functional (GO) classes.

The existence of a positive correlation between the speed of evolution of regulatory sequences and that of coding sequences in orthologous genes is suggestive of a link between rapid diversification of a protein and its expression pattern. We have found that in mammals there is a weak but significant correlation between these two factors, in accordance with previous observations in nematodes [11] and yeast [12]. Interestingly, we have observed that this relationship is especially relevant for non-HK genes, while in HK genes the effect is practically negligible.

The CpG island gene classification and association with expression breadth observed here is consistent with other reports [22,24]. The majority of mammalian promoters contain CpG islands and HK genes are particularly rich in this type of sequence. Our study shows that promoters that do not contain CpG islands are more strongly conserved than those that do, and even more so if the genes encode slowly evolving proteins. Promoters with no CpG islands correspond to classical TATA-containing promoters and it has been recently shown in a large-scale analysis that they are particularly well-conserved across different mammalian species [33].

We identify nine different motifs, corresponding to known transcription factor binding sites, that are significantly over-represented in HK genes. Most of the transcription factors that bind to these sites are themselves encoded by HK genes and the rest are encoded by genes classified as of intermediate expression breadth. Five of the motifs (binding Sp1, USF, NRF1, CREB, or ATF) show high frequency peaks in the vicinity of the TSS (-200 to -1) in a large collection of human promoters, and the combination of two of them (binding Sp1 and NRF1) is over-represented in HK gene promoters [34]. Some of the motifs identified are bound by known regulators of HK genes; examples are Sp1 and USF for the APEX nuclease gene [35] or Sp1 and HIF-1 for the endoglin gene [36].

Of note, besides HK genes, we also find differences between the groups of genes with restricted expression (1-10 tissues) and intermediate expression (11-50 tissues). 'Restricted' genes tend to show higher promoter conservation than 'intermediate' genes (Table 1; Aupplementary Tables S1, S2, and S3 in Additional data file 1). These results may seem counter-intuitive, as one could argue that genes expressed in only a few tissues should have more simple regulation than genes expressed in an intermediate number of tissues. However, one possibility is that 'restricted' genes contain a larger number of negative regulatory elements. Interestingly, gene reporter assays of promoter activity in ENCODE regions (approximately 1% of the genome) have shown that negative elements appear to be present from 1,000 to 500 nucleotides upstream of the TSS in 55% of genes [37]. This indicates that motifs for inhibitory transcription factors may be present in a substantial fraction of genes. One expects that such regions will be more common in tissue-specific 'restricted' genes, which would be consistent with the observed stronger distal promoter sequence conservation.

It has been observed that metazoan-specific proteins tend to be more tissue-specific than universal eukaryotic proteins [20]. In other words, HK genes are enriched for proteins of ancient origin. Old eukaryotic proteins typically evolve more slowly and are longer than proteins of a more recent origin, probably due to increased functional constraints [38]. However, at the level of gene expression regulatory regions they may be simpler and less constrained than genes that represent innovations in multi-cellular organisms. Cross-species comparisons will be used in future studies to gain further insight into these questions.

## Conclusion

We describe that genes with housekeeping expression contain more divergent promoters than genes with a more restricted tissue expression. Importantly, this property cannot be fully explained by the functional class of the encoded gene products, or by a higher prevalence of CpG islands in HK gene promoters. In addition, we have identified a number of transcription factors that are likely to play a predominant role in the control of HK gene expression. We argue that the lower promoter conservation observed in HK genes could be due to a more simple regulation of gene transcription.

## Materials and methods

### Sequence retrieval and alignment

We identified human and mouse orthologous genes using the Ensembl database (release 34) [39]. We considered only orthology relationships of type UBRH (unique best reciprocal hit): 17,620 records of human genes with orthologous mouse genes (human-mouse dataset) and 12,868 of mouse genes with orthologous human genes (mouse-human dataset). We extracted the promoter sequences from these genes, comprising 2 Kb upstream of the TSS, from the UCSC database (hg17 and mm6 releases) [40], excluding genes with multiple TSSs, discarding duplicates, and considering only gene pairs with human-mouse and mouse-human orthology data that were both available and congruent. The resulting dataset contained 8,972 orthologous promoter sequence pairs. We discarded repeats from alignments using RepeatMasker (release 1.1.65) [41]. We aligned the sequences with the local pairwise sequence alignment program described in Castillo-Davis *et al*. [11], using a minimum alignment length of 16 nucleotides. For each orthologous pair we obtained the promoter sequence divergence score (d_SM_; shared motif divergence), which is the fraction of the sequence that does not align, taking the average between the human and mouse promoter sequences. The fraction of sequence aligned was then 1 - d_SM_. We calculated the average 1 - d_SM _in 100 nucleotide sequence windows overlapping by 20 nucleotides. Failure to align portions of the promoter may be due to very high divergence or the occurrence of insertions/deletions. To obtain an estimate of the d_SM _random expectation we aligned, with the same program, 1,000,000 pairs of 2 Kb random sequences and calculated their d_SM _scores. We discarded orthologous pairs with an overall average d_SM _> 0.97 (random expectation ≥0.01), obtaining 7,330 orthologous promoter sequence pairs. Coding sequences were extracted from the Ensembl database (release 34) and aligned with ClustalW [42].

### Substitution rate estimation

Synonymous (Ks) and non-synonymous (Ka) substitution rates were estimated with the codeml program in PAML [13]. From the 7,330 orthologous pairs, 6,698 remained after discarding those with Ka ≥ 0.5, Ks ≥ 2.0, or Kp ≥ 2.0 (saturated pairs). We estimated, for each gene, the number of nucleotide substitutions per site in the concatenated promoter sequence alignment, using the baseml program, with the Hasegawa, Kishino and Yano (1985) model [43], in PAML. This substitution rate was termed Kp.

### Gene expression datasets

We used mouse transcriptome microarray data from Zhang *et al*. [14] to classify the previously defined genes into different groups according to their expression in 55 different mouse organs and tissues (see Supplementary table S5 in Additional data file 1). Zhang *et al*. [14] considered genes to be expressed only if their intensity exceeded the 99th percentile of intensities from the negative controls.

In addition, we used human gene expression data from Gene Atlas (GNF1H), based on transcriptome microarray data [18], and human gene expression data from the eVOC database (anatomical system and cell type ontologies, release 2.7), based on expressed sequence tag data [17]. We considered genes to be expressed in a tissue according to Gene Atlas data only if the expression level was ≥200. Gene Atlas covers 79 human organs and tissues (see Supplementary table S5 in Additional data file 1). For eVOC anatomical systems and cell types we discarded classes with a very small number of genes (<1,000) or large classes with high redundancy (>90% of genes shared with other classes). This resulted in 57 anatomical systems and 10 cell types (see Supplementary table S5 in Additional data file 1). HK, intermediate and restricted expression groups were defined following similar criteria as for the mouse transcriptome data.

Complete sequence divergence data for the different expression groups are available in Additional data file 3.

### Statistical tests and correlations

Correlations were calculated with the Spearman Rank correlation method. Two-sample Wilcoxon-Mann-Whitney statistical test was used to assess differences between groups unless stated. The R statistical package was used [44].

### Gene Ontology functions

GO annotations were extracted from Ensembl (release 34) [39]. We used the GO term definitions of 30 March, 2005 [21]. Over-representation and under-representation of HK genes in different GO classes were verified by chi-square test (*p *< 0.01), using expected values calculated from the percent number of HK genes in the root GO term of each ontology (GO:0003674, molecular function; GO:0008150, biological process; GO:0005575, cellular component). Only GO terms containing a number of genes between 50 and 1,000, both included, were considered. Some GO terms were discarded to reduce redundancies.

### Transcription factor binding site predictions

We used weight matrices from PROMO (release 3) [27,45] and TRANSFAC (release 7.0) [26] to predict transcription factor binding sites. Motif searches were carried out with a similarity cut-off of 0.85. We selected motifs consistently predicted by both matrix collections that were over-represented in HK genes versus all the genes taken together using the chi-square test.

### CpG islands

We extracted sequences -100 to +100 with respect to the TSS. We classified genes as CpG+ (CpG island-positive near TSS), when the C+G content exceeded 0.55 and the CpG score (observed CpG/expected CpG) exceeded 0.65 in the -100 to +100 region, or as CpG- (CpG island-negative near TSS), otherwise. This classification is similar to that used by Yamashita *et al*. [22], but with more stringent values for CpG+ determination, in line with the CpG island definition proposed by Takai and Jones [46]. To study differences in CpG island sequence conservation between HK and non-HK genes, we extended the CpG islands upstream, such that the G+C content exceeded 0.55 and the CpG score exceeded 0.65, calculating in this manner the 5' end point of CpG islands.

## Additional data files

The following additional data are available with the online version of this manuscript. Additional data file 1 contains Supplementary tables S1-S5: Table S1 lists human gene sequence divergence values in expression groups according to Gene Atlas (GNF1H); Table S2 lists human gene sequence divergence values in expression groups according to the eVOC anatomical system classification; Table S3 lists human gene sequence divergence values in expression groups according to the eVOC cell type classification; Table S4 lists GO terms over-represented and under-represented in HK genes with their average d_SM _values; and Table S5 lists the organs, tissues, and cell types considered in each expression dataset. Additional data file 2 contains figures plotting promoter sequence conservation along 2 Kb upstream of the TSS in HK and non-HK genes considering expression groups according to Gene Atlas GNF1H (Figure S1), the eVOC anatomical system classification (Figure S2), and the eVOC cell type classification (Figure S3). Additional data file 3 contains the complete sequence divergence and expression group data used in this manuscript. Additional data file 4 contains human 2 Kb upstream sequences (human promoters), in fasta format. Additional data file 5 contains mouse 2 Kb upstream sequences (mouse promoters), in fasta format.

## Supplementary Material

Additional data file 1 Table S1: human gene sequence divergence values in expression groups according to Gene Atlas (GNF1H). Table S2: human gene sequence divergence values in expression groups according to the eVOC anatomical system classification. Table S3: human gene sequence divergence values in expression groups according to the eVOC cell type classification. Table S4: lists GO terms over-represented and under-represented in HK genes with their average d_SM _values. Table S5: the organs, tissues, and cell types considered in each expression dataset. Click here for file

Additional data file 2 Expression groups are according to Gene Atlas GNF1H (Figure S1), the eVOC anatomical system classification (Figure S2), and the eVOC cell type classification (Figure S3). Click here for file

Additional data file 3 Complete sequence divergence and expression group data used in this manuscript. Click here for file

Additional data file 4 Human 2 Kb upstream sequences (human promoters), in fasta format. Click here for file

Additional data file 5 Mouse 2 Kb upstream sequences (mouse promoters), in fasta format. Click here for file
