# Supplementary material for: Housekeeping genes tend to show reduced upstream sequence conservation
Source: Genome Biol. 2007 Jul 13;8(7):R140. doi: 10.1186/gb-2007-8-7-r140 (PMC2323216; doi:10.1186/gb-2007-8-7-r140)
Supplement: Additional data file 1 — Table S1: human gene sequence divergence values in expression groups according to Gene Atlas (GNF1H). Table S2: human gene sequence divergence values in expression groups according to the eVOC anatomical system classification. Table S3: human gene sequence divergence values in expression groups according to the eVOC cell type classification. Table S4: lists GO terms over-represented and under-represented in HK genes with their average dSM values. Table S5: the organs, tissues, and cell types considered in each expression dataset. [file gb-2007-8-7-r140-S1.pdf]

Additional data file 1

Number of tissues	N *(total=5838)*	d_{SM}	Kp	Ka	Ks	Ka/Ks
01-26	1413	0.687	0.336	**0.096**	**0.745**	**0.135**
		0.729	0.327	0.071	0.676	0.103
		0.217	0.104	0.087	0.317	0.116
27-77	3010	0.695	0.334	0.079	0.699	0.114
		0.749	0.326	0.054	0.630	0.081
		0.223	0.098	0.079	0.302	0.108
78-79	1415	**0.722**	0.329	**0.065**	**0.662**	**0.101**
		0.777	0.327	0.045	0.596	0.074
		0.208	0.088	0.067	0.307	0.100
	p-value (K-W test)	$<10^{-5}$	0.583	$<10^{-27}$	$<10^{-14}$	$<10^{-17}$

Table S1. Sequence divergence values versus Gene Atlas (GNF1H) expression breadth. N: number of genes; d_{SM}: promoter divergence (see text); Kp: promoter substitution rate; Ka: non-synonymous substitution rate; Ks: synonymous substitution rate. Mean (top), median (middle), and standard deviation (bottom) are indicated for each variable. Numbers in bold indicate significant differences at $p < 0.001$ in each expression group with respect to the rest (two-sample Wilcoxon-Mann-Whitney test). Last row shows the p-value of Kruskal-Wallis test that evaluates differences between the three tissue breadth expression groups.

Number of anat. systems	N *(total=6660)*	d_{SM}	Kp	Ka	Ks	Ka/Ks
01-21	1591	**0.677**	0.340	**0.118**	**0.789**	**0.158**
		0.730	0.326	0.093	0.712	0.124
		0.229	0.133	0.098	0.329	0.135
22-41	3295	**0.707**	0.333	**0.084**	**0.714**	**0.122**
		0.763	0.328	0.063	0.638	0.092
		0.215	0.091	0.077	0.307	0.106
42-57	1774	**0.711**	0.330	**0.051**	**0.625**	**0.082**
		0.766	0.326	0.033	0.565	0.059
		0.214	0.084	0.057	0.287	0.084
	p-value (K-W test)	$<10^{-5}$	0.658	$<10^{-142}$	$<10^{-64}$	$<10^{-95}$

Table S2. Sequence divergence values versus eVOC anatomical system expression breadth. N: number of genes; d_{SM}: promoter divergence (see text); Kp: promoter substitution rate; Ka: non-synonymous substitution rate; Ks: synonymous substitution rate. Mean (top), median (middle), and standard deviation (bottom) are indicated for each variable. Numbers in bold indicate significant differences at $p < 0.05$ in each expression group with respect to the rest (two-sample Wilcoxon-Mann-Whitney test). Last row shows the p-value of Kruskal-Wallis test that evaluates differences between the three tissue breadth expression groups.

Number of cell types	N *(total=6195)*	d_{SM}	Kp	Ka	Ks	Ka/Ks
01-02	1339	**0.676**	0.339	**0.109**	**0.765**	**0.152**
		0.731	0.326	0.083	0.691	0.112
		0.230	0.123	0.096	0.322	0.137
03-07	3637	0.705	0.331	0.080	0.700	0.117
		0.762	0.327	0.057	0.628	0.088
		0.216	0.093	0.075	0.302	0.104
08-10	1219	**0.726**	0.331	**0.054**	**0.625**	**0.085**
		0.784	0.326	0.033	0.568	0.060
		0.205	0.084	0.063	0.290	0.085
	p-value (K-W test)	$<10^{-6}$	0.570	$<10^{-77}$	$<10^{-36}$	$<10^{-50}$

Table S3. Sequence divergence values versus eVOC cell type expression breadth. N: number of genes; d_{SM}: promoter divergence (see text); Kp: promoter substitution rate; Ka: non-synonymous substitution rate; Ks: synonymous substitution rate. Mean (top), median (middle), and standard deviation (bottom) are indicated for each variable. Numbers in bold indicate significant differences at $p < 0.0001$ in each expression group with respect to the rest (two-sample Wilcoxon-Mann-Whitney test). Last row shows the p-value of Kruskal-Wallis test that evaluates differences between the three tissue breadth expression groups.

GO TERMS OVER-REPRESENTED IN HOUSEKEEPING GENES		N	% HK	Av. d_{SM}
MOLECULAR FUNCTION				
GO:0003735	Structural constituent of ribosome	54	66.67	**0.77**
GO:0003723	RNA binding	138	57.97	**0.74**
GO:0016874	Ligase activity	124	40.32	0.74
GO:0016817	Hydrolase activity, acting on acid anhydrides	146	37.67	0.72
GO:0000166	Nucleotide binding	464	32.97	0.71
BIOLOGICAL PROCESS				
GO:0043037	Translation	55	74.55	0.74
GO:0006412	Protein biosynthesis	141	59.57	**0.72**
GO:0009117	Nucleotide metabolism	54	57.41	0.71
GO:0006886	Intracellular protein transport	117	52.14	0.72
GO:0051186	Cofactor metabolism	51	50.98	**0.77**
GO:0044249	Cellular biosynthesis	256	49.61	**0.73**
GO:0045184	Establishment of protein localization	162	48.15	0.72
GO:0016070	RNA metabolism	104	47.12	**0.75**
GO:0006512	Ubiquitin cycle	124	44.35	0.72
GO:0006457	Protein folding	59	44.07	0.74
GO:0051243	Negative regulation of cellular physiological process	131	38.17	0.69
GO:0006091	Generation of precursor metabolites and energy	142	38.03	**0.74**
GO:0007049	Cell cycle	188	36.17	0.69
GO:0019538	Protein metabolism	700	36.00	**0.71**
GO:0044260	Cellular macromolecule metabolism	761	35.74	**0.71**
CELLULAR COMPONENT				
GO:0030529	Ribonucleoprotein complex	88	69.32	**0.74**
GO:0005829	Cytosol	78	56.41	0.73
GO:0005739	Mitochondrion	171	54.97	**0.77**
GO:0031090	Organelle membrane	117	52.14	**0.75**
GO:0012505	Endomembrane system	57	43.86	0.74
GO:0005737	Cytoplasm	773	42.95	**0.73**
GO:0005783	Endoplasmic reticulum	153	41.18	**0.73**

GO TERMS UNDER-REPRESENTED IN HOUSEKEEPING GENES		N	% HK	Av. d_{SM}
MOLECULAR FUNCTION				
GO:0004872	Receptor activity	259	8.49	0.69
GO:0015267	Channel or pore class transporter activity	73	12.33	0.66
GO:0004871	Signal transducer activity	440	14.32	**0.66**
GO:0003700	Transcription factor activity	183	18.58	**0.58**
GO:0043169	Cation binding	485	20.21	**0.68**
BIOLOGICAL PROCESS				
GO:0007267	Cell-cell signaling	96	7.29	**0.63**
GO:0030001	Metal ion transport	79	7.59	0.65
GO:0030154	Cell differentiation	78	7.69	**0.57**
GO:0007155	Cell adhesion	141	9.93	**0.64**
GO:0050874	Organismal physiological process	292	10.62	0.69
GO:0009605	Response to external stimulus	209	11.48	0.71
GO:0007166	Cell surface receptor linker signal transduction	221	11.76	**0.64**
GO:0007600	Sensory perception	66	12.12	0.69
GO:0048513	Organ development	214	13.08	**0.59**
GO:0007399	Nervous system development	90	13.33	**0.58**
GO:0009653	Morphogenesis	262	13.36	**0.60**
GO:0009607	Response to biotic stimulus	166	16.87	0.71
GO:0007165	Signal transduction	563	19.89	**0.66**
CELLULAR COMPONENT				
GO:0005576	Extracellular region	219	9.59	**0.64**
GO:0005886	Plasma membrane	373	15.55	**0.67**

Table S4. Gene Ontology (GO) terms over-represented and under-represented in housekeeping genes. N: number of human genes represented in our dataset (N>=50). % HK: percentage of housekeeping genes. d_{SM}: promoter divergence mean. Over-representation and under-representation were verified by χ^2 test ($p<0.01$). GO terms with average d_{SM} values in bold showed a significantly biased d_{SM} distribution ($p<0.01$).

Table S5. Classes (organs, tissues, cell types) of the expression datasets used to estimate expression breadth.

Zhang (organs and tissues)

Adrenal	Heart	Small intestine
Aorta	Hindbrain	Snout
Bladder	Kidney	Spinal cord
Bone Marrow	Knee	Spleen
Brain	Large intestine	Stomach
Brown fat	Liver	Striatum
Calvaria	Lung	Teeth
Cerebellum	Lymph node	Testis
Colon	Mammary gland	Thymus
Cortex	Mandible	Thyroid
Digit	Midbrain	Tongue
E10.5 Head	Olfactory bulb	Tongue surface
E14.5 Head	Ovary	Trachea
ES	Pancreas	Trigeminus
Embryo	Placenta 12.5	Uterus
Embryo 12.5	Placenta 9.5	
Embryo 9.5	Prostate	
Epididymus	Salivary	
Eye	Skeletal Muscle	
Femur	Skin	

Gene Atlas GNF1H

ColorectalAdenocarcinoma	WholeBrain	Pancreas
WHOLEBLOOD	ParietalLobe	PancreaticIslets
BM-CD33+Myeloid	MedullaOblongata	testis
PB-CD14+Monocytes	Amygdala	TestisLeydigCell
PB-BDCA4+Dentritic_Cells	PrefrontalCortex	TestisGermCell
PB-CD56+NKCells	OccipitalLobe	TestisInterstitial
PB-CD4+Tcells	Hypothalamus	TestisSeminiferousTubule
PB-CD8+Tcells	Thalamus	salivarygland
PB-CD19+Bcells	subthalamicnucleus	trachea
BM-CD105+Endothelial	CingulateCortex	AdrenalCortex
BM-CD34+	Pons	Ovary
leukemialymphoblastic(molt4)	spinalcord	Appendix
721_B_lymphoblasts	fetalbrain	skin
lymphomaburkittsRaji	adrenalgland	ciliaryganglion
leukemiapromyelocytic(hl60)	Lung	TrigeminalGanglion
lymphomaburkittsDaudi	Heart	atrioventricularnode
leukemiachronicmyelogenous(k562)	Liver	DRG
thymus	kidney	SuperiorCervicalGanglion
Tonsil	Prostate	SkeletalMuscle
lymphnode	Uterus	UterusCorpus
fetalliver	Thyroid	TONGUE
BM-CD71+EarlyErythroid	fetalThyroid	OlfactoryBulb
bonemarrow	fetallung	Pituitary
TemporalLobe	PLACENTA	
globuspallidus	CardiacMyocytes	
CerebellumPeduncles	SmoothMuscle	
cerebellum	bronchialepithelialcells	
caudatenucleus	ADIPOCYTE	

eVOC (anatomical systems)

bone	ganglion	liver
cartilage	sympathetic chain	bile duct
skeletal muscle	head and neck	kidney
smooth muscle	heart	bladder

dermal system
brain
cerebrum
cerebral cortex
basal nuclei
hypothalamus
medulla oblongata
cerebellum
choroid
retina
optic nerve
cornea
lens
auditory apparatus
peripheral nerve
artery
larynx
lung
bone marrow
blood
tonsil
spleen
tongue
salivary gland
pancreas
pharynx
oesophagus
stomach
small intestine
large intestine
testis
prostate
ovary
breast
umbilical cord
blastocyst
uterus
placenta
endocrine pancreas
pineal gland
pituitary gland
thyroid
parathyroid
adrenal gland
thymus

<u>eVOC (cell types)</u>

squamous cell
transitional cell
lymphoblast
B-lymphocyte
T-lymphocyte
natural killer cell
macrophage
adipocyte
smooth muscle cell
skeletal muscle cell
