# Supplementary material for: Housekeeping genes tend to show reduced upstream sequence conservation
Source: Genome Biol. 2007 Jul 13;8(7):R140. doi: 10.1186/gb-2007-8-7-r140 (PMC2323216; doi:10.1186/gb-2007-8-7-r140)
Supplement: Additional data file 2 — Expression groups are according to Gene Atlas GNF1H (Figure S1), the eVOC anatomical system classification (Figure S2), and the eVOC cell type classification (Figure S3). [file gb-2007-8-7-r140-S2.pdf]

Additional data file 2

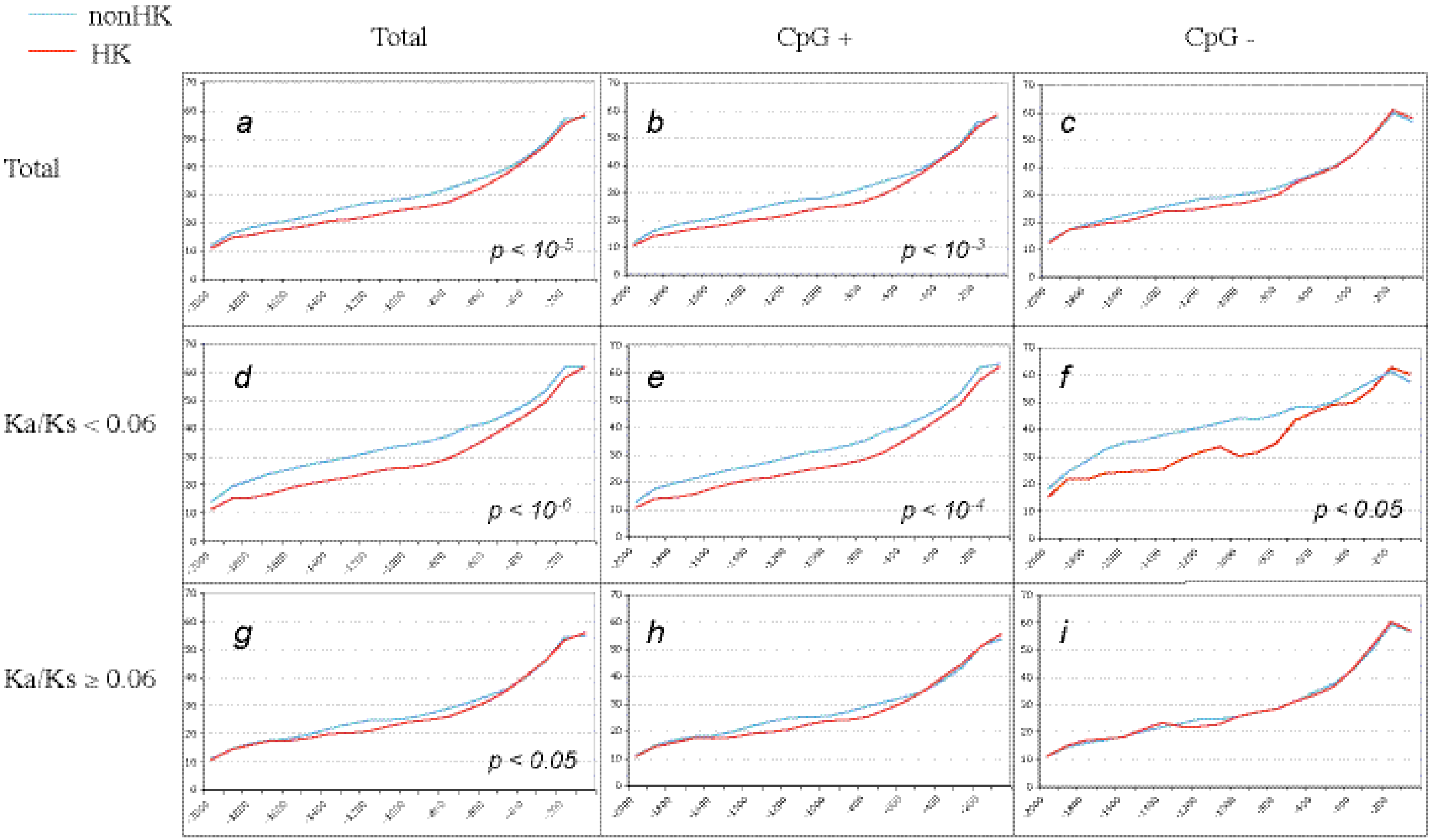

Figure S1. Promoter sequence conservation in housekeeping (HK) and non-housekeeping (non-HK) genes. Expression breadth estimated from Gene Atlas (GNF1H) data. The X-axis shows 100 nucleotide bins along 2Kb upstream of the TSS. The Y-axis shows percent conservation $((1 - d_{SM}) \cdot 100)$. Genes were grouped according to the presence or absence of a CpG island and Ka/Ks values. Significant p-values ($p<0.05$) are indicated.

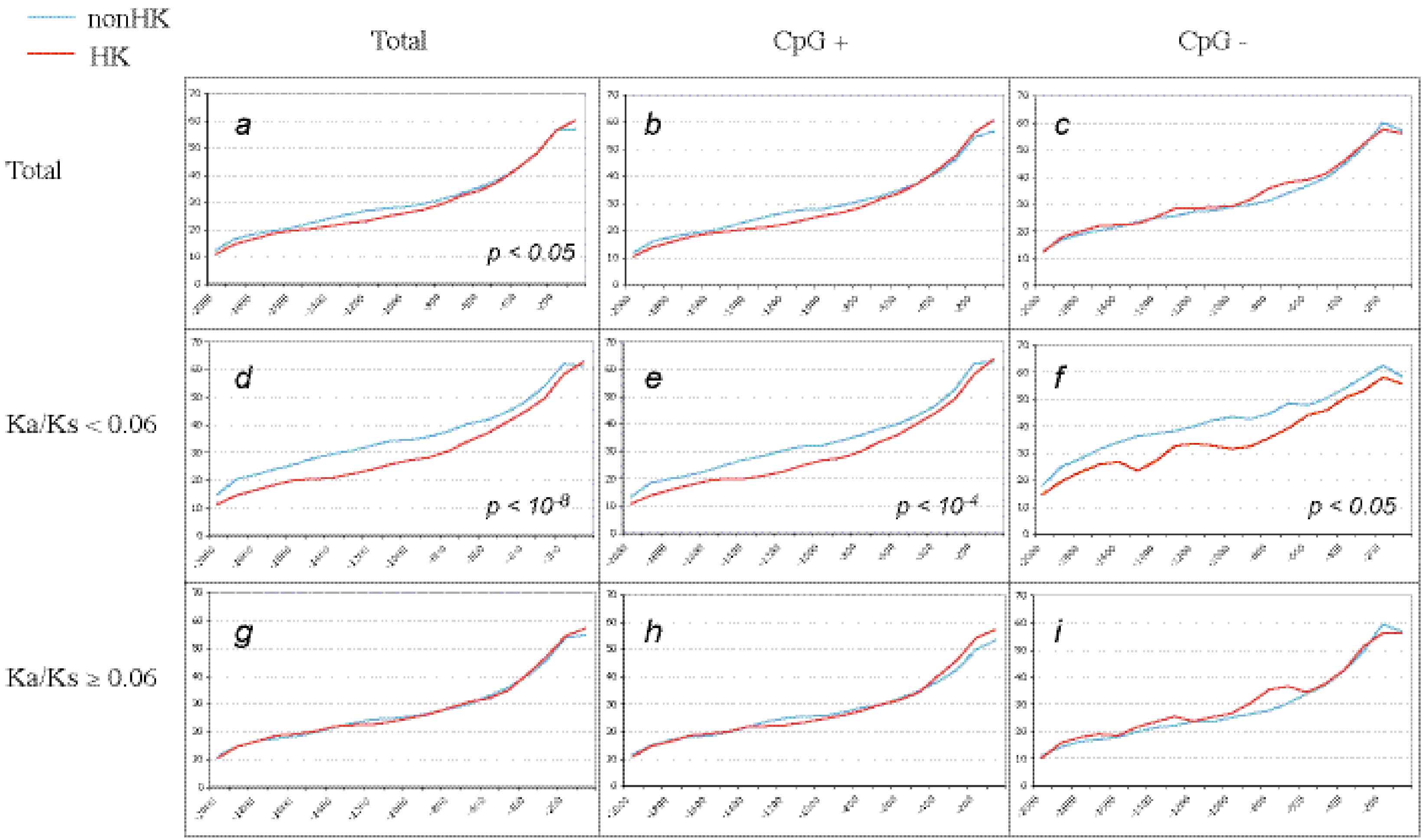

Figure S2. Promoter sequence conservation in housekeeping (HK) and non-housekeeping (non-HK) genes. Expression breadth estimated from eVOC anatomical systems data. The X-axis shows 100 nucleotide bins along 2Kb upstream of the TSS. The Y-axis shows percent conservation ($(1 - d_{SM}) \cdot 100$). Genes were grouped according to the presence or absence of a CpG island and Ka/Ks values. Significant p-values ($p<0.05$) are indicated.

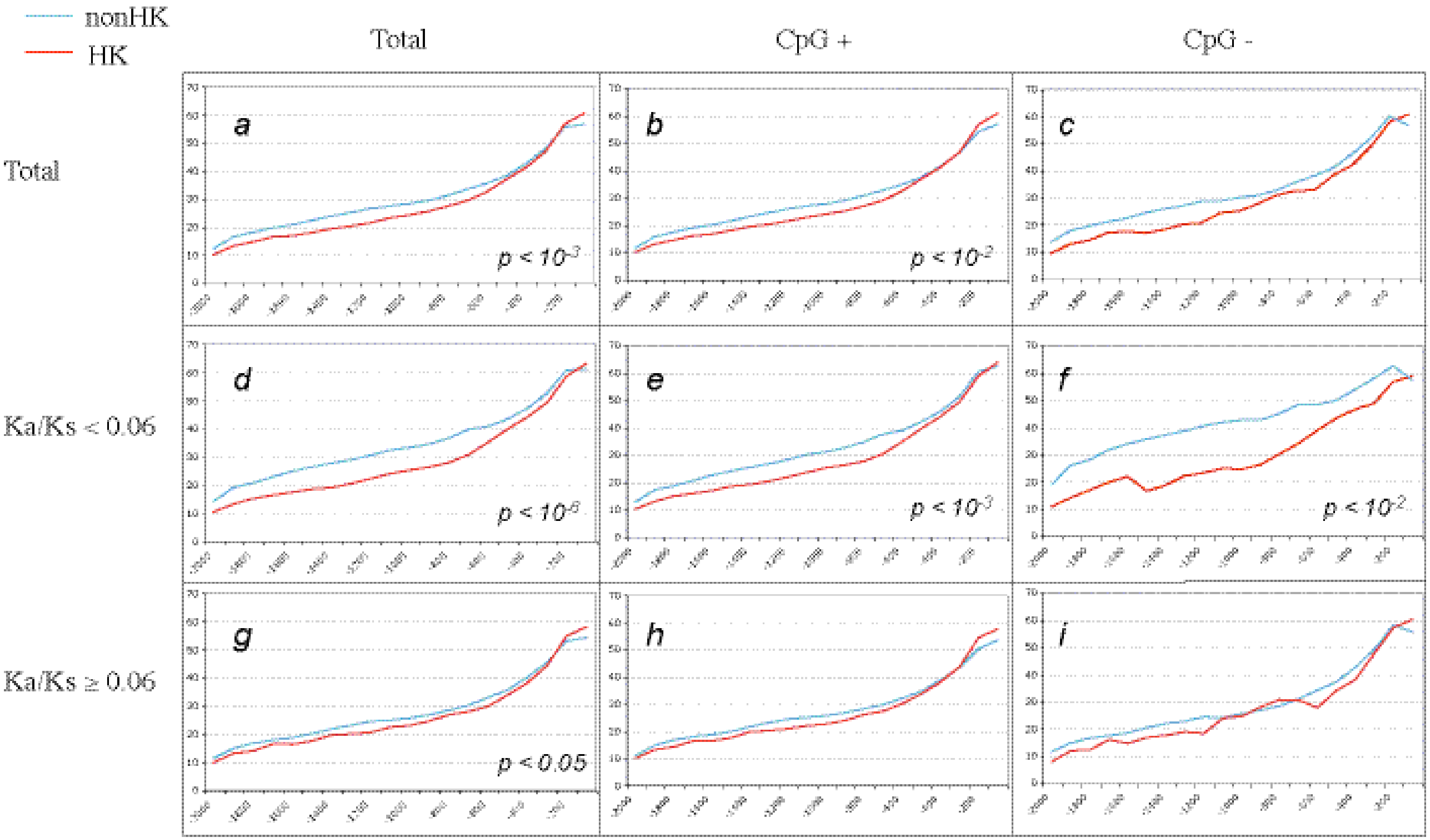

Figure S3. Promoter sequence conservation in housekeeping (HK) and non-housekeeping (non-HK) genes. Expression breadth estimated from eVOC cell types data. The X-axis shows 100 nucleotide bins along 2Kb upstream of the TSS. The Y-axis shows percent conservation ($(1 - d_{SM}) \cdot 100$). Genes were grouped according to the presence or absence of a CpG island and Ka/Ks values. Significant p-values ($p<0.05$) are indicated.
